# Spectroscopy and 1μm Luminescence by Visible Quantum Cutting in Pr^3+^-Yb^3+^ Codoped Glass

**DOI:** 10.3390/ma3042405

**Published:** 2010-03-29

**Authors:** Yumiko Katayama, Setsuhisa Tanabe

**Affiliations:** Graduate School of Human and Environmental Studies, Kyoto university / Yoshida-nihonmatsu-cho, Sakyo-ku, Kyoto 606-8501, Japan; E-Mail: stanabe@gls.mbox.media.kyoto-u.ac.jp (S.T.)

**Keywords:** quantum cutting, praseodymium, ytterbium, glass, solar energy

## Abstract

The quantum cutting phenomenon of a blue photon into two infrared photons is reported in glass codoped with Pr^3+^-Yb^3+^ ions. Oxyfluoride glass with compositions of 32SrF_2_-0.1PrF_3_-2.9YbF_3_-42SiO_2_-23Al_2_O_3_ were prepared, and photoluminescence properties in the range from visible to near-infrared were investigated. Evidence of several energy transfers, such as (Pr^3+^:^3^P_0_→^1^G_4_)→(Yb^3+^:^2^F_5/2_←^2^F_7/2_) and (Pr^3+^:^1^D_2_→^3^F_4_, ^3^F_3_)→(Yb^3+^:^2^F_5/2_←^2^F_7/2_), were demonstrated in the Pr^3+^-Yb^3+^ co-doped glass. By comparing excitation spectrum of the Yb^3+^ emission with absorption spectrum of Pr^3+^, we obtain direct evidence of quantum cutting by excitation to Pr^3+^:^3^P_J_ levels at 420 ~ 490 nm.

## 1. Introduction

The quantum cutting (QC) phosphors that convert a photon into two photons with lower energy have been studied because of their application for such purposes as in fluorescent tubes, plasma display panels and solar cells. In 1974, it was reported that a phosphor doped with Pr^3+^ ions showed QC, which converted a vacuum ultra violet (VUV) photon into 400 nm (^1^S_0_→^1^I_6_,^3^P_J_) and 480 nm (^3^P_0_→^3^H_4_) visible photons [1,2]. Since this report, phosphors that show QC from VUV to visible lights have been reported in materials doped with several rare earth ions and rare earth ions pairs, such as Pr^3+^ [1,2], Gd^3+^ [3], Gd^3+^-Eu^3+^ [4]. These phosphors have been studied as a replacement for the Hg discharge and high quantum efficiency phosphors.

Recently, rare earth ions pairs, RE^3+^-Yb^3+^ (RE = Pr, Tb, Tm) that show visible to near-infrared QC have been reported [5,6,7,8]. These materials have attracted a great attention because they have potential to enhance efficiency of crystalline silicon (c-Si) solar cells [9,10,11]. The solar cells, which are generally based on photovoltaic effect of semiconductor, can obtain electricity from photons that have energy equal to or higher than the bandgap. However, in the case of higher-energy photons, the excess energy of incident photons is changed into heat. This thermal loss is one of the major reasons that photoelectric conversion efficiency of a single-junction solar cell, even with optimum bandgap for the solar spectrum, is limited up to 29% [12]. Among the various solar cells ever developed, the c-Si cell is most widely used because of its many practical advantages. Therefore, it has been reported that spectral modification of the solar spectrum is one solution for further improvement of the conversion efficiency. Since the Yb^3+^:^2^F_5/2_→^2^F_7/2_ emission and sensitivity peak of silicon solar cell overlap each other, these QC materials, which convert a photon in the UV to blue region into 1.2 eV-photons (λ = 1 μm), would be an ideal phosphor for the c-Si cells.

In this study, we prepared oxyfluoride glass doped with Pr^3+^ and Yb^3+^ ions. Generally, glass material can be a preferred solution for solar cell applications, because it is transparent in wide wavelength regions, can be used as a cover material and thus can easily substitute for the conventional ones already used widely for the installed modules.

In this paper, the absorption, emission and excitation spectra of the glass were investigated. We examined energy transfer efficiency between Pr^3+^ and Yb^3+^ ions, particularly from Pr^3+^:^3^P_J_ and ^1^D_2_ to the Yb^3+^-excited level by in-depth consideration of the emission spectra obtained. Comparing excitation spectra of Yb^3+^, emission and absorption spectra, as well as energy transfer efficiency, we obtain direct evidence of quantum cutting in the oxyfluoride glass.

## 2. Experimental

A Pr^3+^-Yb^3+^ codoped glass with compositions of 32SrF_2_-0.1PrF_3_-2.9YbF_3_-42SiO_2_-23Al_2_O_3_ and Pr^3+^-Gd^3+^ codoped glass with compositions of 32SrF_2_-xPrF_3_-(3-x)GdF_3_-42SiO_2_-23Al_2_O_3_ (x = 0.1, 1) were prepared using SrF_2_, PrF_3_, YbF_3_, GdF_3_, SiO_2_, Al_2_O_3_ as raw materials. Since the Gd^3+^ ion is optically inert in the wavelength range of visible to infrared, we will call the Pr^3+^-Gd^3+^ codoped glass as “Pr^3+^ singly doped glass”. All chemical powders had 99.9−99.99% purity. After mixing well in an alumina mortar, 20 g batches were melted in a platinum crucible at 1350 °C for 1.5 h. The melts were poured onto a stainless-steel plate at room-temperature and pressed by another plate. The obtained glass was annealed for 1 h at 500 °C below the glass transition temperature, which was determined by a differential scanning calorimeter, DSC (Rigaku, Thermo plus, DSC8270). The absorption spectrum was measured by using two kinds of absorption spectrometers (Shimadzu UV3600 and FT-IR8400S). The emission spectra in the range of 450−1200 nm were measured with a computer-controlled monochromator (Nikon, G-250) and a Si photodiode (Electro-Optical System Inc., S-025-H) by pumping with a 440 nm laser diode and a 590 nm light monochromatized by a bandpass filter (ASAHI SPECTRA, XBPA590) from a Xenon light source (ASAHI SPECTRA, MAX-302). Luminescence excitation spectrum was measured by using the Xenon light source, the monochromator and the Si photodiode with an 850 nm short-cut filter.

## 3. Results

**Figure 1:**
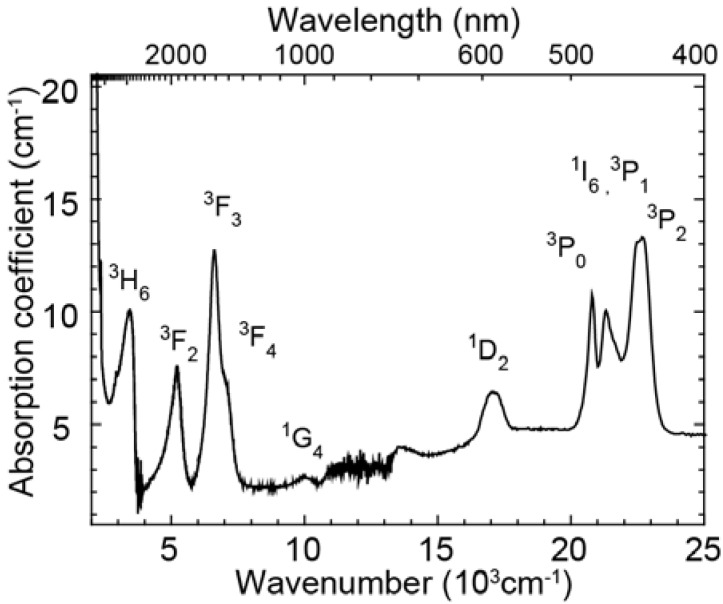
Absorption spectrum of the Pr^3+^ singly doped oxyfluoride glass (x = 1.0).

Figure 1 shows the absorption spectrum of the glass doped with Pr^3+^ ions. The absorption bands of excited levels, ^3^H_6_, ^3^F_2_, ^3^F_3_, ^3^F_4_, ^1^G_4_, ^1^D_2_, ^3^P_0_, ^3^P_1_, ^1^I_6_, ^3^P_2_ were observed. Overlapping bands around 7000 cm^-1^ and 23,000 cm^-1^ were deconvoluted into two Gaussian bands by least-square fitting and the energy level values in the glass sample were determined. These absorption peak energy values were used to calculate the energy of Pr^3+^ emissions and to carry out the peak assignments. The Pr^3+^ singly doped glass has no strong absorption in the range of 500 nm ~ 1100 nm where c-Si solar cells have high conversion efficiency.

**Figure 2:**
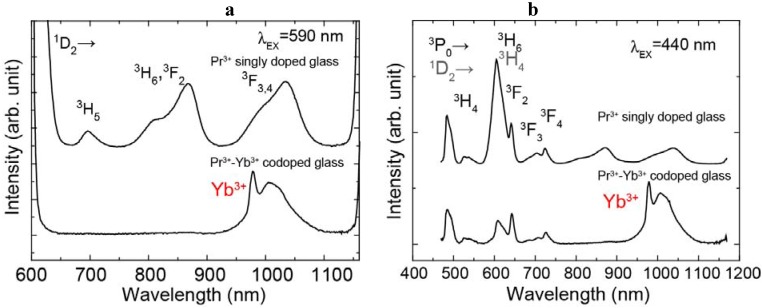
Emission spectra of the Pr^3+^ singly doped and Pr^3+^-Yb^3+^ codoped samples excited at (a) 590 nm and (b) 440 nm.

**Figure 3:**
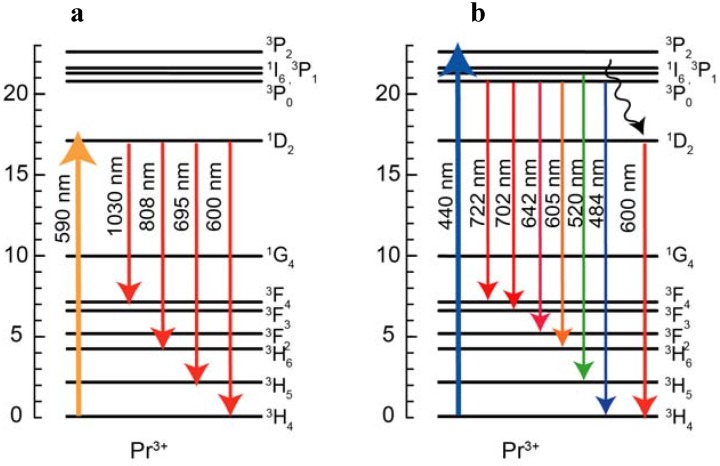
Energy level diagram of Pr^3+^ with transitions excited at (a) 590 nm and (b) 440 nm.

The emission spectra of the Pr^3+^ singly doped and the Pr^3+^-Yb^3+^ codoped samples excited at 440 nm and 590 nm light are shown in Figure 2 (a) and (b). The energy diagram of Pr^3+^ ion and transitions are shown in Figure 3. The emission spectrum of the Pr^3+^ singly doped sample excited at 590 nm showed 695 nm, 808 nm, 1030 nm emissions and these emissions originate from the ^1^D_2_→^3^H_5_, ^1^D_2_→^3^F_2_ and ^1^D_2_→^3^F_3, 4_ transitions, respectively. In the emission spectrum of the Pr^3+^ singly doped sample excited at 440 nm, we observed several emissions from Pr^3+^:^3^P_1_ and ^3^P_0_ levels, 484 nm (^3^P_0_→^3^H_4_), 520 nm (^3^P_1_→^3^H_4_), 605 nm (^3^P_0_→^3^H_6_), 642 nm (^3^P_0_→^3^F_2_), 702 nm (^3^P_0_→^3^F_3_), 722 nm (^3^P_0_→^3^F_4_) and also observed emissions from the ^1^D_2_.

The Pr^3+^-Yb^3+^ codoped sample showed Yb^3+^ (^2^F_5/2_→^2^F_7/2_) emission excited at both 440 nm and 590 nm. Under excitation at 440 nm, the intensity of the Yb^3+^ emission of the Pr^3+^-Yb^3+^ codoped sample was about twice as large as the Pr^3+^ emissions. We also found that the Pr^3+^ emission peak around 600 nm of the Pr^3+^-Yb^3+^ codoped sample is quite different from that of the singly doped one. Under excitation at 590 nm, we cannot observe Pr^3+^ emissions in the Pr^3+^-Yb^3+^ codoped sample.

**Figure 4:**
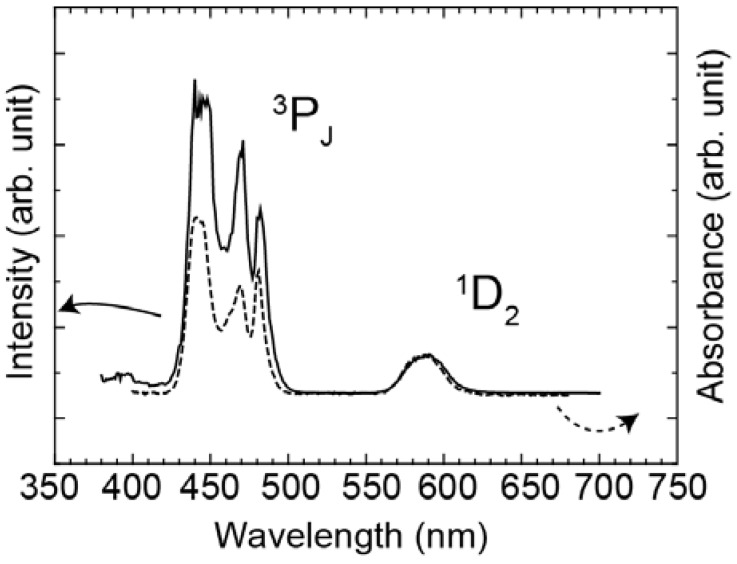
Excitation spectrum of Yb^3+^ 1μm luminescence of the Pr^3+^-Yb^3+^ codoped glass (solid line) and absorption spectrum of the Pr^3+^ doped glass (dotted line).

Figure 4 shows the excitation spectrum of Yb^3+^ 1μm luminescence of the Pr^3+^-Yb^3+^ codoped glass and the absorption spectrum of the Pr^3+^ singly doped glass. The excitation cross-section ratio of the ^1^D_2_ to the ^3^P_J_ band was approximately 1:10 and the absorption cross-section ratio was approximately 1:6.

## 4. Discussion

In the codoped sample, the Pr^3+^ concentration is so low (0.1 mol %) that cross relaxation and energy migration processes between Pr^3+^ ions can be neglected. As indicated in Figure 2 (b), the Pr^3+^ singly doped glass showed the Pr^3+^:^1^D_2_ emissions. This indicates that multi-phonon relaxation from ^3^P_0_ to ^1^D_2_ occurs in the sample. Taking into account the multi-phonon relaxation from ^3^P_0_ to ^1^D_2_, we find that the emission band around 600 nm is a convolution of two bands, due to transitions of ^3^P_0_→^3^H_6_ and ^1^D_2_→^3^H_4_.

**Figure 5:**
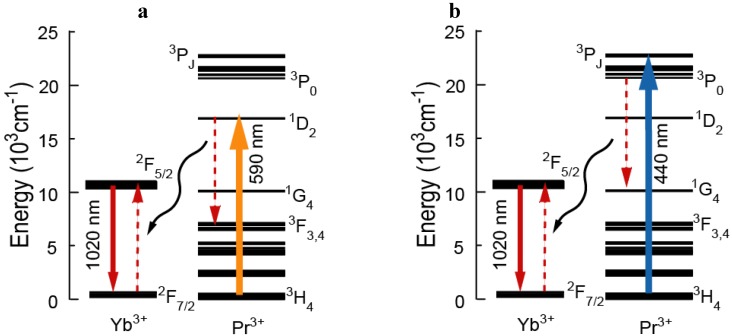
Energy transfer mechanisms of Pr^3+^ and Yb^3+^ excited at (a) 590 nm and (b) 440 nm.

Yb^3+^ emissions were observed in the Pr^3+^-Yb^3+^ codoped glass excited at both 440 nm and 590 nm. This result indicates two energy transfers (ET) from Pr^3+^ to Yb^3+^ ions: (Pr^3+^:^3^P_0_→^1^G_4_) →(Yb^3+^:^2^F_5/2_←^2^F_7/2_) and (Pr^3+^:^1^D_2_→^3^F_4_, ^3^F_3_)→(Yb^3+^:^2^F_5/2_←^2^F_7/2_), as shown in Figure 5.

Now, we discuss ET efficiency (η_ET_) from Pr^3+^ to Yb^3+^ ions in the Pr^3+^-Yb^3+^ codoped sample. ET efficiency (η_ET_) can be expressed by the following equation (1),

(1) $$\eta_{ET}=\frac{W_{ET}}{A+W_{MP}+W_{ET}}$$

where A is radiative transition, W_MP_ is multi-phonon relaxation and W_ET_ represents ET rate. Since the radiative transitions from Pr^3+^:^3^P_0_ level were observed as indicated in Figure 2 (b), ET efficiency from Pr^3+^:^3^P_0_ to Yb^3+^, η_ET_ (^3^P_0_) is less than 100%. On the other hand, as indicated in Figure 2 (a), no radiative transition of Pr^3+^:^1^D_2_ was observed. Since almost all of ^1^D_2_ emissions were quenched, ET efficiency from Pr^3+^:^1^D_2_ to Yb^3+^, η_ET_ (^1^D_2_) is close to 100%. Therefore, the η_ET_ (^1^D_2_) is higher than the η_ET_ (^3^P_0_). The radiative transition, A, for ^3^P_0_ is very likely to be higher than for ^1^D_2_, as the radiative lifetime of the former level is usually shorter than for the latter. This is one reason why the η_ET_ (^1^D_2_) is higher than the η_ET_ (^3^P_0_). It is likely that in the present system W_MP_(^1^D_2_) is negligible, due to the relatively large energy gap, and for ^1^D_2_ W_ET_ is larger than A; this makes η_ET_ (^1^D_2_) close to 100%. For ^3^P_0_, W_MP_ is more important due to the smaller gap, and also A is higher, making η_ET_ (^3^P_0_) less than 100%.

**Figure 6:**
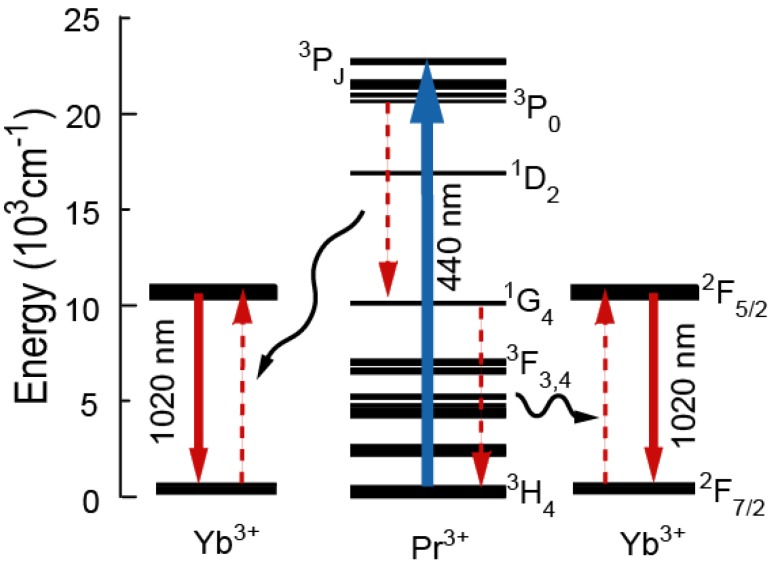
Schematic diagram of quantum cutting between Pr^3+^ and Yb^3+^ ions.

Excitation spectrum monitoring the Yb^3+^ emission and the absorption spectrum are shown in Figure 4. Area ratio of the ^1^D_2_ band to the ^3^P_J_ band in the excitation spectrum was 1:10, while that of the absorption spectrum was 1:6. When we assume that excitation efficiency to Yb^3+^ from ^3^P_J_ and that from ^1^D_2_ are equivalent, the emission intensity of Yb^3+^ ions by excited ^3^P_J_ levels is more than 1.6 times as strong as that of the ^1^D_2_ level. In fact, there is the following relation between the ^3^P_J_ band and the ^1^D_2_ band: EX (^3^P_J_)/EX (^1^D_2_)>1.6, where EX (^3^P_J_) and EX (^1^D_2_) are the excitation efficiency of ^3^P_J_ and ^1^D_2_, respectively. This is direct evidence of quantum cutting as indicated in Figure 6. In the case of the ET processes shown in Figure 5, ETs from both ^1^D_2_ and ^3^P_0_ of Pr^3+^ to Yb^3+^ are one photon to one photon processes. As previously mentioned, the ET efficiency from ^1^D_2_ (η_ET_ (^1^D_2_)) is higher than that from ^3^P_0_ (η_ET_ (^3^P_0_)). If the ET occurs as shown in Figure 5, the excitation efficiency ratio of Yb^3+^, EX (^3^P_J_)/EX (^1^D_2_) is less than unity. Thus, the one photon-one photon ET process cannot explain the experimental results. There is the possibility of a cooperative three body energy transfer between one excited Pr^3+^ and two Yb^3+^ ions, as shown in Figure 6. The ideal one photon to two photon process, EX (^3^P_J_)/EX (^1^D_2_) = 2 can be achieved in the case of low nonradiative loss due to slow multi-phonon relaxation from ^3^P_0_ and ^1^G_4_.

## 5. Conclusions

The Pr^3+^-Yb^3+^ codoped oxyfluoride glass showed Yb^3+^ emissions when excited at Pr^3+^:^3^P_J_ and ^1^D_2_ indicating two schemes of ET: (Pr^3+^:^3^P_0_→^1^G_4_)→(Yb^3+^:^2^F_5/2_←^2^F_7/2_) and (Pr^3+^:^1^D_2_→^3^F_4_, ^3^F_3_)→(Yb^3+^:^2^F_5/2_←^2^F_7/2_). Comparing the Yb^3+^ excitation spectrum to the absorption spectrum, we find that the Yb^3+^ excitation efficiency by ^3^P_J_, EX(^3^P_J_) is 1.6 times greater than EX(^1^D_2_), which indicates that two step ETs of (Pr^3+^:^3^P_0_→^1^G_4_)→(Yb^3+^:^2^F_5/2_←^2^F_7/2_) and ET(Pr^3+^:^1^G_4_→^3^H_4_)→(Yb^3+^:^2^F_5/2_→^2^F_7/2_) occur in the Pr^3+^-Yb^3+^ codoped glass when excited at 440 nm. This is the direct evidence of quantum cutting. The direct evidence of quantum cutting in a glass sample supports its promising application to improve the solar cell efficiency.
